# Modelling the overdiagnosis of breast cancer due to mammography screening in women aged 40 to 49 in the United Kingdom

**DOI:** 10.1186/bcr3365

**Published:** 2012-11-29

**Authors:** Necdet B Gunsoy, Montserrat Garcia-Closas, Sue M Moss

**Affiliations:** 1 Division of Genetics and Epidemiology, Institute of Cancer Research, 15 Cotswold Road, Sutton, SM2 5NG, UK; 2 Breakthrough Breast Cancer Centre, Institute of Cancer Research, 237 Fulham Road, London, SW3 6JB, UK; 3 Centre for Cancer Prevention, Queen Mary University of London, Wolfson Institute of Preventive Medicine, Charterhouse Square, London, EC1M 6BQ, UK

## Abstract

**Introduction:**

Overdiagnosis of breast cancer due to mammography screening, defined as the diagnosis of screen-detected cancers that would not have presented clinically in a women's lifetime in the absence of screening, has emerged as a highly contentious issue, as harm caused may question the benefit of mammographic screening. Most studies included women over 50 years old and little information is available for younger women.

**Methods:**

We estimated the overdiagnosis of breast cancer due to screening in women aged 40 to 49 years using data from a randomised trial of annual mammographic screening starting at age 40 conducted in the UK. A six-state Markov model was constructed to estimate the sensitivity of mammography for invasive and *in situ *breast cancer and the screen-detectable mean sojourn time for non-progressive *in situ*, progressive *in situ*, and invasive breast cancer. Then, a 10-state simulation model of cancer progression, screening, and death, was developed to estimate overdiagnosis attributable to screening.

**Results:**

The sensitivity of mammography for invasive and *in situ *breast cancers was 90% (95% CI, 72 to 99) and 82% (43 to 99), respectively. The screen-detectable mean sojourn time of preclinical non-progressive and progressive *in situ *cancers was 1.3 (0.4 to 3.4) and 0.11 (0.05 to 0.19) years, respectively, and 0.8 years (0.6 to 1.2) for preclinical invasive breast cancer. The proportion of screen-detected *in situ *cancers that were non-progressive was 55% (25 to 77) for the first and 40% (22 to 60) for subsequent screens. In our main analysis, overdiagnosis was estimated as 0.7% of screen-detected cancers. A sensitivity analysis, covering a wide range of alternative scenarios, yielded a range of 0.5% to 2.9%.

**Conclusion:**

Although a high proportion of screen-detected *in situ *cancers were non-progressive, a majority of these would have presented clinically in the absence of screening. The extent of overdiagnosis due to screening in women aged 40 to 49 was small. Results also suggest annual screening is most suitable for women aged 40 to 49 in the United Kingdom due to short cancer sojourn times.

## Introduction

Since the introduction of mammography screening in many countries, a substantial increase in the incidence of breast cancers has been observed, raising concern about the potential for overdiagnosis of breast cancer due to screening. However, no consensus has been reached on the extent of such overdiagnosis. An overdiagnosed breast cancer is defined as one which is screen-detected, and would never have presented clinically in a woman's lifetime in the absence of screening [1]. In addition to overdiagnosis and consequent overtreatment, screening results in additional years lived with breast cancer due to the advancement of time of diagnosis. Estimates of overdiagnosis in previous studies vary considerably. Comparisons of expected breast cancer incidence extrapolated from rates before the introduction of screening with that observed after have resulted in estimates of overdiagnosis ranging from 4% [2] to 52% [3] of all diagnosed breast cancers. Variations between estimates reflect the methodological challenges faced when estimating overdiagnosis. A drop in breast cancer incidence is observed in the age group immediately above that invited for screening due to the advancement of diagnosis of these cases by screening. Studies that do not account for this compensatory drop tend to have a higher estimate of overdiagnosis. Estimates will also vary depending on whether or not *in situ *cancers are included [1,4].

Simulation modelling is a popular tool for estimating the extent of overdiagnosis due to screening; it requires estimates of the mean duration of pre clinical cancer states (mean sojourn time), the screening test sensitivity (STS), and the background incidence of breast cancer in the absence of screening. De Koning *et al*. applied this approach to Dutch screening data for women aged 50 to 74, and estimated that 3% of all cancers and 8% of screen-detected cancers were overdiagnosed [5].

Most estimates of overdiagnosis are based on data for women aged 50 years and over, as younger women are currently not eligible for screening in most countries. The extension of the age range of screening programmes to include younger women is under debate, but little is known on the extent of overdiagnosis due to screening in these women. Also, evidence suggest that younger women tend to have breast cancers that progress faster and lower mammography STS, mostly due to higher breast density, than older women [6-9], which may be favourable with regards to overdiagnosis.

In this study, we model data from a trial of mammographic screening for breast cancer starting at age 40 (Age trial) conducted in the UK, using data collected from the start of the trial in 1991 until 31st December 2010, in order to estimate, in women aged 40 to 49:

1. the STS of mammography for invasive and *in situ *breast cancers, that is the probability of a mammographic screen detecting a cancer that is in the preclinical state,

2. the mean sojourn time (MST), that is the mean duration, in years, for a cancer from first becoming detectable by screening to clinical diagnosis, of the screen-detectable preclinical breast cancer states: progressive *in situ*, non-progressive *in situ*, and invasive,

3. the proportion of screen-detected *in situ *cancers that are non-progressive,

4. the proportion of breast cancers diagnosed that would not have presented clinically in the absence of screening after accounting for a compensatory drop in incidence.

## Materials and methods

### Data

Details of the Age trial are given elsewhere [10]. In summary, a randomised controlled trial was designed to assess the effectiveness of annual screening by mammography in women from age 40 onwards in the UK. The trial comprised of an intervention arm of 53,890 women assigned to annual screening invitation and a control arm of 106,971 women not offered screening. Recruitment began in 1991 and the trial included 23 centres. Women were invited each year, except for those who specified that they did not wish to participate in the trial. Two-view mammography was performed on the first attendance to screening. The following screens were single view unless otherwise indicated. All diagnosed breast cancers, including interval, screen-detected, and those in the control arm, were recorded and submitted to a pathologic review.

Women with diagnosed breast cancer at entry to the trial were excluded from this analysis. Out of the 53,890 women assigned to the intervention arm, 36,348 attended the first screen and were eligible for analysis. For each following screen, women were included only if they attended all previous screening rounds. Only screening episodes recorded from ages 40 to 49 years as part of the trial were considered, and the analysis was limited to the first eight screening invitations since few women received more than this number. The exact date of each screen and of any breast cancer diagnosis was known for each woman. Interval cancers were defined as cancers diagnosed up to 12 months after a negative routine screen, and time since the previous negative screen was calculated in months. In all eight screening rounds, a total of 194 screen-detected, and 122 interval cancers were recorded. The numbers of women screened, and of screen-detected and interval cancers in each screening round are presented for *in situ *and invasive cancers in Table 1.

**Table 1:** Cancer detection by screening round in a trial of annual mammographic screening starting age 40, UK.

Screening round	Women screened^1 ^	Screen-detected cancers		ICs^2 ^in the first 12 months		Mean age (years)	Mean screening interval (years)
		**INV**^3^	***IS***^4^	**INV**^3^	***IS***^4^		

1	36 348	31	6	7	2	40.5	-

2	30 779	20	3	17	2	41.6	1.1

3	27 083	16	3	17	0	42.6	1.1

4	24 188	15	5	17	1	43.7	1.1

5	21 107	16	4	10	0	44.7	1.0

6	18 603	13	7	16	1	45.6	1.0

7	17 193	19	9	18	1	46.6	1.0

8	13 998	21	6	11	2	47.5	0.9

**Total**	189 299	151	43	113	9	43.4	

^1^Only includes women screened at all previous rounds; ^2^interval cancer; ^3^invasive carcinomas; ^4^*in-situ *carcinomas.

The Age trial is registered as an International Standard Randomised Controlled Trial, number 24647151. Ethical approval was obtained for the trial from London MREC (MREC/98/2/40), and NGIB (formerly PIAG) approval (PIAG 3-07(h)/2002) was obtained for the use of identifiable patient information.

### Statistical methods

**Figure 1:**
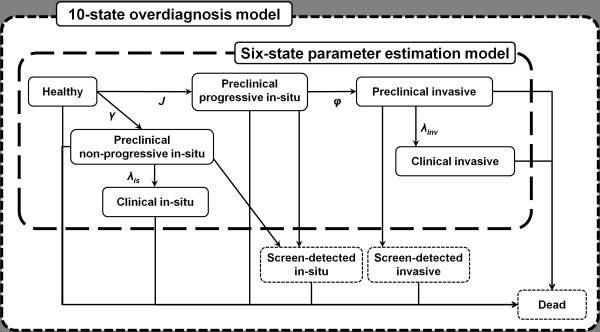
**Graphical respesentation of a six-state parameter estimation model and a 10-state overdiagnosis model**. This figure shows states included in the parameter estimation model, a six-state Markov model of breast cancer progression for estimating screening parameters including screening test sensitivity and mean sojourn time, and the overdiagnosis model, an extended 10-state model aimed at estimating the extent of overdiagnosis. Dotted states are those added in the extension from the former to the latter model. The screen-detected *in situ *state groups two distinct states: screen-detected progressive and non-progressive *in situ*.

We constructed two Markov models to estimate the extent of overdiagnosis (Figure 1): one to estimate screening parameters based on the Age trial data (parameter estimation model), and one to estimate overdiagnosis based on parameter estimates from the first model (overdiagnosis model).

#### Parameter estimation model

We constructed a six-state Markov model similar to that in a previous study [11]; states included were healthy, screen-detectable non-progressive *in situ *(NPIS), clinically diagnosed non-progressive *in situ *(CIS), screen-detectable progressive *in situ *(PIS), screen-detectable preclinical invasive breast cancer (PIBC), and clinically diagnosed invasive breast cancer (CIBC).

Several important assumptions were made on the natural history of breast cancer in order to simplify the model specification. First, we assumed that there were two types of *in situ *lesions: one that would progress into invasive cancer, and another that would never progress into invasive cancer, but could become clinically detected in the absence of screening. Thus, PIS would not be diagnosed in the absence of screening and would eventually progress into PIBC before becoming clinically diagnosed. Also, *in situ *cancers detected at screening would be either from PIS or NPIS, whereas those observed in the screening interval or in the absence of screening would be exclusively from NPIS. Second, we assumed that all PIBC have a mandatory PIS precursor. Finally, we assumed that preclinical cancers could not regress, but only remain in their current state or progress.

The intensity matrix, *Q*, for this model, with *J*, the background incidence of invasive breast cancer, *γ*, the background incidence of *in situ *breast cancer, *ϕ*, the transition rate between PIS and PIBC, *λ_is_*, the transition rate between NPIS and CIS, and *λ_inv_*, the transition rate between PIBC and CIBC was determined as

(1) $$Q=\begin{array}{cc} & STATE\\ 1 & \text{Healthy}\\ 2 & \text{NPIS}\\ 3 & \text{PIS}\\ 4 & \text{PIBC}\\ 5 & \text{CIBC}\\ 6 & \text{CIS} \end{array}\left(\begin{array}{cccccc} 1 & 2 & 3 & 4 & 5 & 6\\ -\left(\gamma +J\right) & \gamma & J & 0 & 0 & 0\\ 0 & -\lambda_{is} & 0 & 0 & 0 & \lambda_{is}\\ 0 & 0 & -\phi & \phi & 0 & 0\\ 0 & 0 & 0 & -\lambda_{inv} & \lambda_{inv} & 0\\ 0 & 0 & 0 & 0 & 0 & 0\\ 0 & 0 & 0 & 0 & 0 & 0 \end{array}\right).$$

Both *J *and *γ *were obtained directly from the observed age-specific incidence in the control arm of the Age trial who were not offered any screening between the ages of 40 to 49 years. Given the assumption of exponential distribution of time to transition, the MST in a state was the inverse of the transition rate. From the intensity matrix, the probability of progression from any state *i *to any state *j *in any time interval *t*, can be defined as *P_ij _*(*t*). The derivation of transition probabilities is based on the solution of Kolmogorov equations and exponential distribution properties and will not be developed here [11-13]. Given transition probabilities, *P_ij _*(*t*), the probability of having a positive or negative mammogram, as well as the incidence of breast cancer in the interval between two screens can be formulated (Table 2).

**Table 2:** Probability of cancer detection at prevalent and incident screens, and monthly incidence of interval cancers.

Description	Parameter	Definition
**Any screen ***k *		

Number of women screened	n.scr*_k_*	see Table 1
Negative screening episodes	n.neg*_k_*	$\text{n}\text{.sc}{\text{r}}_{k}\left(1-\text{P}\text{.sc}{{\text{r}}_{npis}}_{{}_{k}}-\text{P}\text{.sc}{{\text{r}}_{pis}}_{{}_{k}}-\text{P}\text{.sc}{{\text{r}}_{inv}}_{{}_{k}}\right)$

**Proportion of false negatives at screen ***k*		

False negative NPIS	$\text{P}\text{.fne}{{\text{g}}_{npis}}_{{}_{k}}$	$\left(\text{P}\text{.sc}{\text{r}}_{npis_{k}}\text{n}\text{.sc}{\text{r}}_{k}\left(1-S_{is}\right)/S_{is}\right)/\text{n}\text{.ne}{\text{g}}_{k}$
False negative PIS	$\text{P}\text{.fne}{{\text{g}}_{pis}}_{{}_{k}}$	$\left(\text{P}\text{.sc}{\text{r}}_{pis_{k}}\text{n}\text{.sc}{\text{r}}_{k}\left(1-S_{is}\right)/S_{is}\right)/\text{n}\text{.ne}{\text{g}}_{k}$
False negative PIBC	$\text{P}\text{.fne}{{\text{g}}_{inv}}_{{}_{k}}$	$\left(\text{P}\text{.sc}{\text{r}}_{inv_{k}}\text{n}\text{.sc}{\text{r}}_{k}\left(1-S_{inv}\right)/S_{inv}\right)/\text{n}\text{.ne}{\text{g}}_{k}$
Any pre-cancer state	$\text{P}\text{.fne}{\text{g}}_{k}$	$\text{P}\text{.fne}{{\text{g}}_{npis}}_{{}_{k}}+\text{P}\text{.fne}{{\text{g}}_{pis}}_{{}_{k}}+\text{P}\text{.fne}{\text{g}}_{inv_{k}}$

**Prevalent screen **		

Screen-detected NPIS	$\text{P}\text{.sc}{{\text{r}}_{npis}}_{{}_{1}}$	*S_is_P*_12_(age)/(*P*_11_(age) + *P*_12_(age) + *P*_13_(age) + *P*_14_(age))
Screen-detected PIS	$\text{P}\text{.sc}{{\text{r}}_{pis}}_{{}_{1}}$	*S_is_P*_13_(age)/(*P*_11_(age) + *P*_12_(age) + *P*_13_(age) + *P*_14_(age))
Screen-detected PIBC	$\text{P}\text{.sc}{{\text{r}}_{inv}}_{{}_{1}}$	*S_inv_P*_14_(age)/(*P*_11_(age) + *P*_12_(age) + *P*_13_(age) + *P*_14_(age))

**Incident screen ***k *		

Screen-detected NPIS	$\text{P}\text{.sc}{{\text{r}}_{npis}}_{{}_{k}}$	$S_{is}\left(\left(1-\text{P}\text{.fne}{\text{g}}_{k-1}\right){\text{P}}_{12}\left(t\right)+\text{P}\text{.fne}{\text{g}}_{npis_{k-1}}P_{22}\left(t\right)\right)$
Screen-detected PIS	$\text{P}\text{.sc}{{\text{r}}_{pis}}_{{}_{k}}$	$S_{is}\left(\left(1-\text{P}\text{.fne}{\text{g}}_{k-1}\right){\text{P}}_{12}\left(t\right)+\text{P}\text{.fne}{\text{g}}_{npis_{k-1}}P_{22}\left(t\right)\right)$
Screen-detected PIBC	$\text{P}\text{.sc}{\text{r}}_{inv_{k}}$	$S_{inv}\left(\left(1-\text{P}\text{.fne}{\text{g}}_{k-1}\right)P_{14}\left(t\right)+\text{P}\text{.fne}{\text{g}}_{pis_{k-1}}P_{34}\left(t\right)+P.\text{fne}{\text{g}}_{inv_{k-\text{1}}}P_{44}\left(t\right)\right)$

**Monthly incidence of interval cancers in the first ***m ***months after screen ***k*		

In-situ	$\text{I}{\text{C}}_{is_{k}}$	$\begin{array}{c} \text{n}\text{.ne}{\text{g}}_{k}\left(P_{11}\left(m-1\right)P_{16}\left(1\right)+P_{12}\left(m-1\right)P_{26}\left(1\right)\right)\\ +\text{P}\text{.sc}{\text{r}}_{is_{k}}\text{n}\text{.sc}{\text{r}}_{k}\left(1-S_{is}\right)/S_{is}\left(P_{22}\left(m-1\right)P_{26}\left(1\right)\right) \end{array}$
Invasive	$\text{I}{\text{C}}_{inv_{k}}$	$\begin{array}{c} \text{n}\text{.ne}{\text{g}}_{k}\left(P_{11}\left(m-1\right)P_{15}\left(1\right)+P_{13}\left(m-1\right)P_{35}\left(1\right)+P_{14}\left(m-1\right)P_{45}\left(1\right)\right)\\ +\text{P}\text{.sc}{\text{r}}_{inv_{k}}\text{n}\text{.sc}{\text{r}}_{k}\left(1-S_{inv}\right)/S_{inv}\left(P_{44}\left(m-1\right)P_{45}\left(1\right)\right)\\ +\text{P}\text{.sc}{\text{r}}_{pis_{k}}\text{n}\text{.sc}{\text{r}}_{k}\left(1-S_{is}\right)/S_{is}\left(P_{34}\left(m-1\right)P_{45}\left(1\right)+P_{33}\left(m-1\right)P_{35}\left(1\right)\right) \end{array}$

Age, age at first screen; NPIS, non-progressive *in situ *breast cancer; PIBC, preclinical invasive breast cancer; PIS, progressive *in situ *breast cancerl; t, screening interval.

Women in this analysis were free from diagnosed breast cancer at entry, meaning that, at the prevalent screen, probabilities were conditional on being healthy or in a preclinical disease state. The number of women screened in each round, n.scr*_k_*, is given in Table 1. The STS for *in situ *and invasive cancer were defined as *S_is _*and *S_inv_*, respectively. In each screen, we defined the probability of screen detection of NPIS, PIS, and PIBC. The model was fitted to the observed number of *in situ *and invasive cancers detected in each screen. For incident screens, we defined the probability of having a false negative result in the previous screen for each preclinical cancer state. The monthly incidence of interval cancers was defined for CIS and CIBC and fitted to the observed incidence of *in situ *and invasive cancers, respectively, in the first 12 months after each screen. This analysis was performed using WinBUGS14. The median and 95% credible interval (CI) of the posterior distribution for each parameter was obtained through Gibbs sampling, using 5 chains of 3000 iterations. Due to their correlated nature, we also estimated the correlation between MST and STS.

### Overdiagnosis model

The first model was extended to include 10 states in order to estimate the absolute amount of overdiagnosis due to screening (Figure 1). States included healthy, preclinical NPIS, preclinical PIS, PIBC, screen-detected NPIS, screen-detected PIS, CIS, screen-detected PIBC, CIBC, and dead. In the simulation, 1,000,000 women were followed up for 15 years in monthly cycles starting from age 40. Transitions probabilities between states were calculated using data from various sources: (1) the Office for National Statistics (ONS), for the incidence of invasive and *in situ *breast cancer [14] and all-cause death rates [15] in women aged 40 to 54 from 2008, (2) the Age trial [10], for the incidence of invasive and *in situ *breast cancer in women aged 40 to 49, (3) the parameter estimation model in this study, for the STS and MST in pre-cancer states in women aged 40 to 49, and (4) estimates from previous studies [7,8,11] for the MST in women aged over 50. For the breast cancer incidence in women aged 40 to 44, we used incidence rates reported by ONS. For women aged 44 to 49, ONS incidence rates are affected by screening at age 49; we therefore used the incidence in the control arm of the Age trial, adjusted by the ratio of the ONS rates to Age trial rates for the 40 to 44 age group. This resulted in higher incidence rates than those observed in the Age trial control arm; it was therefore not necessary to adjust these rates for selection bias due to the lower observed rate in non-attenders. For the base-case analysis, the medians of our MST and STS estimates were used. We also performed sensitivity analyses to investigate the impact of changing MST and STS on the estimate of overdiagnosis. We considered the following scenarios: high MST, low MST, high STS, low STS, high MST with low STS, and high MST with high STS (Table 3). This analysis was performed using TreeAge Pro 2011 (TreeAge Software Inc., Williamstown, MA, USA).

**Table 3:** Parameter definitions for base-case and sensitivity analyses of overdiagnosis model.

	Screening test		Mean sojourn time (years)					
	**sensitivity (%)**		**40 to 49**			**50-59**		

Invasive	**Invasive**	**In-situ**	**NPIS**	**PIS**	**PIBC**	**NPIS**	**PIS**	**PIBC**

Base case	Median^1^		Median			3	0.175	1.825

Long MST	Median		Upper limit^2^			5	0.25	2.75

Short MST	Median		Lower limit^3^			2	0.1	1.5

High sensitivity	100	100	Median			3	0.175	1.825

Low sensitivity	Lower limit		Median			3	0.175	1.825

Low sensitivity, long MST	Lower limit		Upper limit			5	0.25	2.75

High sensitivity, long MST	100	100	Upper limit			5	0.25	2.75

^1,2,3^Refers to the median, upper limit, and lower limit of results from parameter estimation model, respectively. MST, median sojourn time; NPIC, non-progressive in situ; PIBC, preclinical invasive breast cancer; PIS, preclinical progressive in situ.

## Results

Estimates from the parameter estimation model are shown in Table 4. The median and 95% CI for the invasive and *in situ *mammography STS were 90.0% (72.0 to 98.9) and 81.7% (43.4 to 99.0), respectively. Model estimates for the MST in the screen-detectable PIBC state was 0.84 years (0.64 to 1.21), which, added to the MST in the screen-detectable PIS state, 0.11 years (0.05 to 0.19), gave a mean window of 0.95 years for a cancer to be detected via screening before arising clinically. For screen-detectable NPIS, the MST was 1.29 years (0.41 to 3.44). The estimated proportion of screen-detected *in situ *cancers that were non-progressive was 55% (25-77) in the prevalent and 40% (22 to 60) in incident screens.

**Table 4:** Model estimates of breast cancer screening and progression parameters in women aged 40 to 49 years

Parameter	Estimate	
	**Median**	**95% CI**

**Screening test sensitivity (%)**		

Invasive	90.0	(72.0-98.9)

In-situ	81.7	(43.4-99.0)

**Mean sojourn time (years)**		

Invasive	0.84	(0.64-1.21)

Progressive *in situ*	0.11	(0.05-0.19)

Non-progressive *in situ*	1.29	(0.41-3.44)

**% of screen-detected *in situ *that is non-progressive**		

Prevalent screen	55	(25-77)

Incident screens	40	(22-60)

Results of the overdiagnosis model are given in Table 5. In our base-case analysis, 16,030 breast cancers were diagnosed between the ages of 40 and 49 years in women offered screening, in contrast with 15,425 in women not offered any screening, a surplus of 605 cases, equivalent to 6.2% of screen-detected and 3.8% of all cases. However, in ages 50 to 54, where screening is not offered in both simlulated groups, 541 additional cases were diagnosed in women not offered screening previously, resulting in a total of 64 overdiagnosed cases equivalent to 0.7% of screen-detected cases and 0.4% of all cancers diagnosed within ages 40 to 49 years.

**Table 5:** Comparison of the number of cancers detected for 1,000,000 women in annual screening between ages 40 to 49 years versus no screening versus.

	Ages 40 to 49		Ages 50 to 54		Ages 40 to 54	
	**Group 1 Annual screening**	**Group 2 No screening**	**Group 1 No screening**	**Group 2 No screening**	**Group 1 Total**	**Group 2 Total**

**Base-case**						

**Screen-detected**						
Invasive	7772	-	-	-	7772	-
Progressive *in situ *	1145	-	-	-	1145	-
Non-progressive *in situ*	874	-	-	-	874	-

**Clinically detected**						
Invasive	5693	14 089	7680	8151	13 373	22 240
Non-progressive *in situ *	546	1336	658	728	1204	2064

***All cancers ***	16 030	15 425	8338	8879	24 368	24 304

**Overdiagnosis**						

Absolute number	605		-541		64	

% of screen-detected	6.2				0.7	

% of cancers diagnosed within ages 40 to 49	3.8				0.4	

Estimates from the overdiagnosis model.

Estimates of overdiagnosis in our sensitivity analysis ranged from 0.5 to 2.9% of screen-detected cancers and 0.3% to 2.2% of all cancers diagnosed within ages 40 to 49 years. The highest impact on overdiagnosis was observed when increasing the MST, whereas increasing the STS had a smaller impact on overdiagnosis (Table 6).

**Table 6:** Overdiagnosis of breast cancer due to annual screening in women aged 40 to 49 years.

Overdiagnosis		
**Scenario**	**% of screen-detected **	**% diagnosed within ages 40 to 49 **

Base-case	0.7	0.4

Long MST	2.7	2.0

Short MST	0.5	0.3

High sensitivity	0.7	0.4

Low sensitivity	0.5	0.3

High sensitivity, long MST	2.9	2.2

Low sensitivity, long MST	2.7	1.6

Results of sensitivity analysis. Overdiagnosis

### Model fit

When compared to data from the Age trial, the parameter estimation model accurately predicted the screen-detected invasive cancers for the first six screens, but underestimated those for the last two screens (Table 7). For screen-detected *in situ *cancers, model predictions were accurate for the first five screens, but underestimated the observed number for the last three screens. The number of invasive interval cancers were overestimated for the first screen, and underestimated in the last three screens. The expected number of *in situ *interval cancers were underestimated for the last screen only. For all screens combined, the model slightly underestimated the number of screen-detected invasive cancers. Expected values from the overdiagnosis models were within the range of estimates from the parameter estimation model, and had a similar fit to the observed data in the intervention arm. The fit of the expected numbers of cancers in the control arm was good, with a slight overprediction overall of approximately 2%.

**Table 7:** Fit of model estimates to data observed in a trial of annual mammographic screening starting age 40 in the UK.

	Invasive			In-situ		
	**Observed **	**Expected**		**Observed **	**Expected**	

**Screening episode **	**AGE trial **	**Paremeter estimation model **	**Overdiagnosis model **	**AGE trial **	**Paremeter estimation model **	**Overdiagnosis model **

		**Intervention arm (offered annual screening) **				

		**Screen-detected cancers **				

**1 **	31	28(22-34)	32	6	7(3-12)	7

**2 **	20	18(16-20)	19	3	5(2-8)	5

**3 **	16	17(15-19)	18	3	5(2-7)	5

**4 **	15	17(15-18)	17	5	5(2-6)	5

**5 **	16	15(13-17)	16	4	5(2-6)	4

**6 **	13	14(13-15)	15	7	3(2-6)	4

**7 **	19	14(12-15)	14	9	3(2-6)	4

**8 **	21	11(10-12)	12	6	3(2-4)	3

**Total **	151	134(116-150)	143	43	36(17-55)	37

**Interval cancers **						

**1 **	7	19(16-23)	16	2	1.4(0.6-2.6)	2

**2 **	17	17(14-19)	14	2	1.3(0.5-2.4)	1

**3 **	17	15(13-18)	13	0	1.2(0.5-2.2)	1

**4 **	17	13(11-15)	13	1	1(0.4-1.9)	1

**5 **	10	12(10-14)	12	0	0.9(0.4-1.7)	1

**6 **	16	12(10-13)	11	1	0.9(0.4-1.7)	1

**7 **	18	10(8-11)	11	1	0.7(0.3-1.4)	1

**8 **	11	4(3-5)	9	2	0.2(0.1-0.5)	1

**Total **	113	102(85-118)	99	9	7.6(3.2-14.4)	9

**Control arm (not offered screening) **						

**40 **	52	-^1^	69	1	-	5

**41 **	115	-	111	8	-	7

**42 **	115	-	124	7	-	9

**43 **	129	-	133	7	-	12

**44 **	138	-	147	15	-	13

**45 **	161	-	155	16	-	12

**46 **	161	-	160	12	-	10

**47 **	165	-	165	7	-	11

**48 **	172	-	166	13	-	12

**Total **	1208	-	1230	86	-	91

^1^The parameter estimation model was built using the incidence in the control arm.

## Discussion

In this study, we aimed to quantify the overdiagnosis of breast cancer attributable to screening women aged 40 to 49 years annually by first estimating screening parameters in a six-state Markov model using data from a trial of annual mammographic screening starting age 40 conducted in the UK. In women aged 40 to 49 years in the UK, we estimated that only 0.3% to 2.2% of all cancers were overdiagnosed.

An implicit assumption of the Markov process is that the time to transition is distributed exponentially. This distribution has been used in many instances previously and has been shown to have a good fit to progression models for breast cancer, as well as other cancers [11,12,16]. We assumed that all *in situ *cancers that arose in the absence of screening were non-progressive. In reality, a proportion of *in situ *cases detected in the absence of screening may be progressive. However, this proportion could not be estimated when relaxing this assumption.

Estimates from our overdiagnosis model suggest a 5% reduction in the detection of invasive cancers as a result of screening. Previously, three screening trials (the Two-County, Stockholm, and Goteborg trials) showed a non-significant reduction of 5% to 10% in the incidence of invasive breast cancer when comparing the incidence in the screened and control groups [17]. Also, we assumed that all invasive cancers had an *in situ *precursor, which may not be the case [18]. Hence, our model may have overestimated the number of invasive cancers detected in their *in situ *precursor state. However, it is unlikely that this would affect our estimates of overdiagnosis as both *in situ *and invasive breast cancers were included in our calculations.

Our results showed that the STS for *in situ *cancers was approximately 10% lower than for invasive cancers, probably due to the larger size of invasive tumours [19]. Although no previous studies estimated the STS of *in situ *cancers separately, previous estimates of the STS for preclinical breast cancer ranged from 69% to 100% [6-8,13], consistent with our 90% STS estimate for PIBC. In the Age trial, two-view mammograms were performed at prevalence screen, and one-view at incident screens unless indicated otherwise. When estimated separately, we found a 10% difference in prevalent (95%, 79 to 100) and incident STS (85%, 69 to 95). However, this model did not show any improvement in fit, and could not estimate the difference in STS for *in situ *and invasive breast cancer, which are more important parameters with regards to overdiagnosis due to screening. In addition, the STS of mammography is likely to increase with increasing age [9]; it was not possible to incorporate this in the current model, but our sensitivity analysis found that increasing the sensitivity had limited impact on the estimate of overdiagnosis. Estimates of MST and STS are necessarily related. The correlation between STS and MST in our model was 0.75 and their distribution showed no sign of bimodality. The sensitivity analysis addressed the correlated nature of STS and MST, by the inclusion of a scenario with long MST and small STS as an alternative to our base-case model, which had short MST and high STS.

The MST for screen-detectable NPIS was the longest among pre-cancer states, suggesting that more NPIS are detected in the prevalent screen than in incident screens. For screen-detectable PIS, the MST was very short, roughly three to ten weeks. Being much shorter than the yearly screening interval, this implies that few progressive lesions are detected in the *in situ *stage. However, the pool of progressive lesions will renew itself at each screen, implying that the rate of progressive lesions detected at each screen is constant proportionally to the background incidence of invasive breast cancer. According to our estimates, the combined MST of PIS and PIBC was under one year in 66% of cases. This would support annual screening for women aged 40 to 49. Biennial or triennial screening would result in many women developing both pre-cancer and having a clinical diagnosis during the screening interval.

To our knowledge, only one study used a six-state Markov model to estimate the detection rates of NPIS in screening, but did not estimate STS [11]. Using UK data for women aged 50 and over, authors predicted that 39% and 21% of screen-detected *in situ *cancers were non-progressive at prevalent and incident screens, respectively. The model had a lack of fit for UK data, overestimating cancers detected at prevalent and underestimating cancers detected at incident screens. In this study, we predicted a higher proportion of NPIS, possibly due to a higher relative background incidence of *in situ *to invasive cancer in women aged 40 to 49 compared to women aged 50 to 69 [14]. In previous studies, the MST of PIBC in women aged 40 to 49 ranged from 1.05 to 2.46 years [6-8,13,20], which is longer than our estimate of 0.95 years. However, this study is the first to report MST estimates for women aged 40 to 49 in the UK, and for older women, previous estimates show a shorter MST in British women compared to other European countries [11].

Our estimate of overdiagnosis for annual screening in women aged 40 to 49 in the UK was in line with those reported in other studies. Hellquist *et al*. [21] estimated that 1% (-6 to 8) of all breast cancers were overdiagnosed in a screening programme for women aged 40 to 49 screened every 18 months in Sweden. In a systematic review, the range of overdiagnosis for women aged 40 to 49 years was -4% to 7.1% [22]. Despite large credible intervals in our estimates of STS and MST, the range of overdiagnosis from this study was small, 0.3% to 2.2% of breast cancers diagnosed within ages 40 to 49 years. Thus, although precise estimates of STS and MST are hard to obtain, the estimate of overdiagnosis is relatively unaected. Our sensitivity analysis included a large range of STS and MST values, and our results should be generalisable to other countries with similar breast cancer incidence rates as the UK. However, it is not clear to what extent our results are extendable to programmes with longer screening intervals: the impact of screening frequency on overdiagnosis in women aged 40 to 49 years would require further studies.

## Conclusions

The most important implication of this study is that, in women aged 40 to 49 in the UK, a small proportion of breast cancers were overdiagnosed due to screening, between 0.3% to 2.2% of all breast cancers diagnosed within ages 40 to 49 years. Since women aged 40 to 49 have shorter MST, lower STS, and lower mortality rates than women aged 50 and over, less overdiagnosis would normally be expected which may explain why estimates of overdiagnosis from this study are smaller than those reported for women aged 50 onwards [2,3,23,24]. Second, although a high proportion of *in situ *cancers detected at screening were estimated to be non-progressive, the great majority of these would have presented clinically in the absence of screening, implying they would not be overdiagnosed. Finally, the mean sojourn time of preclinical invasive breast cancer, including its *in situ *precursor, was just under one year, suggesting that annual screening would be most appropriate for women aged 40 to 49.

## Abbreviations

CIBC: clinical invasive breast cancer; CIS: clinical *in situ*; MST: mean sojourn time; NPIS: non-progressive *in situ*; PIBC: preclinical invasive breast cancer; PIS: preclinical progressive *in situ*; STS: screening test sensitivity.

## Competing interests

The authors declare that they have no competing interests.

## Authors' contributions

NBG participated in the conception and design of the study, the development of the methodology and the interpretation of results, performed the analyses, and drafted the manuscript. MGC participated in the conception of the study, the interpretation of results and reviewed the manuscript. SMM participated in the conception and design of the study, the acquisition of data, the development of the methodology and the interpretation of results, revised and reviewed the manuscript, and supervised the study. All authors read and approved the final manuscript.
